# Small Molecule Inhibitors Confirm Ubiquitin-Dependent Removal of TOP2-DNA Covalent Complexes[Fn FN4]

**DOI:** 10.1124/mol.119.118893

**Published:** 2020-09

**Authors:** Rebecca L. Swan, Luke L.K. Poh, Ian G. Cowell, Caroline A. Austin

**Affiliations:** Newcastle University Biosciences Institute, Newcastle University, Newcastle upon Tyne, United Kingdom

## Abstract

DNA topoisomerase II (TOP2) is required for the unwinding and decatenation of DNA through the induction of an enzyme-linked double-strand break (DSB) in one DNA molecule and passage of another intact DNA duplex through the break. Anticancer drugs targeting TOP2 (TOP2 poisons) prevent religation of the DSB and stabilize a normally transient intermediate of the TOP2 reaction mechanism called the TOP2-DNA covalent complex. Subsequently, TOP2 remains covalently bound to each end of the enzyme-bridged DSB, which cannot be repaired until TOP2 is removed from the DNA. One removal mechanism involves the proteasomal degradation of the TOP2 protein, leading to the liberation of a protein-free DSB. Proteasomal degradation is often regulated by protein ubiquitination, and here we show that inhibition of ubiquitin-activating enzymes reduces the processing of TOP2A- and TOP2B-DNA complexes. Depletion or inhibition of ubiquitin-activating enzymes indicated that ubiquitination was required for the liberation of etoposide-induced protein-free DSBs and is therefore an important layer of regulation in the repair of TOP2 poison–induced DNA damage. TOP2-DNA complexes stabilized by etoposide were shown to be conjugated to ubiquitin, and this was reduced by inhibition or depletion of ubiquitin-activating enzymes.

**SIGNIFICANCE STATEMENT:**

There is currently great clinical interest in the ubiquitin-proteasome system and ongoing development of specific inhibitors. The results in this paper show that the therapeutic cytotoxicity of DNA topoisomerase II (TOP2) poisons can be enhanced through combination therapy with ubiquitin-activating enzyme inhibitors or by specific inhibition of the BMI/RING1A ubiquitin ligase, which would lead to increased cellular accumulation or persistence of TOP2-DNA complexes.

## Introduction

DNA topoisomerase II (TOP2) mediates important changes in DNA topology that are essential for processes, such as chromosome condensation, chromosome segregation, replication, and transcription (Nitiss, 2009a; Pommier et al., 2016). These enzymes catalyze a “strand passage” mechanism whereby one double-stranded DNA molecule is passed through a double-stranded break in another. TOP2 forms an intermediate enzyme-bridged DNA gate termed the TOP2-DNA covalent complex (or cleavage complex), wherein each monomer of the dimeric TOP2 molecule is covalently bound to one end of the double-strand break (DSB) through a 5′-phosphotyrosyl bond. After strand passage, the break is religated, and TOP2 dissociates from DNA. As the DSB is covalently coupled to and buried within the TOP2 enzyme, DNA cleavage does not initiate the DNA damage response that is generally observed after the appearance of DSBs (Mårtensson et al., 2003).

The ability of TOP2 to induce DSBs is exploited in cancer therapy through the use of TOP2 poisons which inhibit the religation of the enzyme-induced DSB and lead to the persistence of DSBs concealed by TOP2-DNA covalent complexes (Nitiss, 2009b). DNA repair requires the liberation of the DSB, which occurs upon the removal of TOP2 protein from the TOP2-DNA complex (Mårtensson et al., 2003). TOP2-DNA covalent complexes can be removed through proteasomal degradation of TOP2 (Mao et al., 2001; Zhang et al., 2006; Fan et al., 2008; Lee et al., 2016), leaving behind a residual phosphotyrosyl peptide adduct that can then be removed by the 5′-phosphodiesterase, TDP2 (Cortes Ledesma et al., 2009; Zeng et al., 2011; Schellenberg et al., 2012; Gao et al., 2014). Alternatively, stabilized TOP2-DNA complexes can be processed in a nuclease-dependent pathway involving Mre11 (of the MRN complex), which may be stimulated by CtIP (Neale et al., 2005; Hartsuiker et al., 2009; Hamilton and Maizels, 2010; Nakamura et al., 2010; Lee et al., 2012; Aparicio et al., 2016; Hoa et al., 2016; Wang et al., 2017). Other proteasome-independent mechanisms of TOP2-DNA complex processing have also been described, including the direct removal of TOP2 by TDP2 in cooperation with the ZATT SUMO ligase (Schellenberg et al., 2016, 2017). Inactivation of TDP2 does not significantly affect the processing of TOP2-DNA complexes to DSBs in proteasome-inhibited cells, suggesting the majority of TOP2-DNA complexes are removed by pathways other than the TDP2/ZATT-dependent pathway (Lee et al., 2018).

There are two TOP2 isoforms in human cells [DNA topoisomerase II*α* (TOP2A) and II*β* (TOP2B)], and both form stabilized TOP2-DNA complexes in the presence of TOP2 poisons (Willmore et al., 1998). Earlier publications suggested that TOP2B complexes are preferentially degraded (Mao et al., 2001; Isik et al., 2003; Azarova et al., 2007). However, later papers have demonstrated that TOP2A is also degraded by the proteasome in response to TOP2 poisons, including etoposide, teniposide, and mitoxantrone (Zhang et al., 2006; Fan et al., 2008; Alchanati et al., 2009; Lee et al., 2016). This was demonstrated both by Western blot (Fan et al., 2008; Alchanati et al., 2009) and through direct measurement of TOP2-DNA complexes using the In Vivo Complex of Enzyme (ICE) assay (Fan et al., 2008) and Trapped in Agarose DNA Immunostaining (TARDIS) assay (Sunter et al., 2010; Lee et al., 2016). The half-life of TOP2B-DNA complexes is shorter than that of TOP2A (Willmore et al., 1998; Errington et al., 2004; Lee et al., 2016), which may account for the perceived “preferential degradation” of TOP2B. The processing of TOP2-DNA complexes can also be investigated through the measurement of TOP2 poison–induced DSBs. As alluded to above, DSBs buried within TOP2-DNA complexes do not themselves elicit a DNA damage response in the form of histone H2A family member X (H2AX) phosphorylation unless the complexes are processed to protein-free DSBs. Indeed, TOP2 poison–induced S-139 phospho-histone H2AX (*γ*H2AX) levels (and other markers of DNA damage) are reduced by cotreatment of cells with a proteasome inhibitor (Zhang et al., 2006; Fan et al., 2008; Tammaro et al., 2013), which is consistent with a role for the proteasome in the liberation of protein-free DSBs from TOP2-DNA complexes.

Proteasomal degradation often (but not always) requires the ubiquitination of the target protein, which is catalyzed by a ubiquitin-activating enzyme (UAE). Two studies investigating the requirement for UAE in the processing of TOP2-DNA complexes have yielded conflicting results. Although both studies employed the same murine cell line ts85 containing a temperature-sensitive UAE, growth at the nonpermissive temperature curtailed TOP2 poison–induced depletion of TOP2B in the first study but did not affect TOP2 poison–induced depletion of TOP2B in the second study (Mao et al., 2001; Ban et al., 2013). Thus, both ubiquitin-dependent and ubiquitin-independent mechanisms for TOP2B protein degradation have been hypothesized. In this study we seek to clarify the requirement of ubiquitination for the processing of TOP2-DNA covalent complexes. This was investigated by inactivation of UAE using a combination of small interfering RNA (siRNA) knockdown and small molecule inhibitor approaches. MLN7243 is a potent E1 inhibitor that forms an MLN7243-ubiquitin adduct, thus leading to inhibition of both UAE enzymes in human cells (UAE1 and UBA6) (Misra et al., 2017; Hyer et al., 2018).

Previous studies have mainly used Western blotting techniques to study the degradation of TOP2 upon etoposide or teniposide treatment. Here we use the TARDIS assay to investigate the effect of UAE inhibition. TARDIS is an immunofluorescence-based technique that visualizes covalently bound TOP2-DNA complex levels in individual cells in a quantifiable manner. This contrasts with other techniques, such as the In Vivo Complex of Enzyme assay, which examines pooled cell populations. The processing of TOP2-DNA complexes was also investigated using the *γ*H2AX assay to measure the appearance of TOP2-free DSBs. We show that UAE activity is required for the efficient removal of both TOP2A and TOP2B complexes from DNA and the subsequent appearance of TOP2-free DSBs, indicating a ubiquitin-dependent processing pathway.

## Materials and Methods

### 

#### Cell Culture and Reagents.

K562 cells, the human pre-B cell line Nalm-6, and the *TOP2B−/−* derivatives of Nalm-6 were grown in RPMI medium containing 10% FBS and 5% penicillin-streptomycin (%v/v) and incubated at 37°C, 5% CO_2_. MLN7243 [TAK-243, sulfamic acid, ((1R,2R,3S,4R)-2,3-dihydroxy-4-((2-(3-((trifluoromethyl)thio)phenyl)pyrazolo(1,5-a)pyrimidin-7-yl)amino)cyclopentyl)methyl ester] (Hyer et al., 2018) was purchased from Active Biochem (Hong Kong). PRT4165 (Ismail et al., 2013) (2-pyridin-3-ylmethylene-indan-1,3-dione) was purchased from Merck Millipore, MA. UAE1 and UBA6 siRNA were purchased from ThermoFisher Scientific (MA, siRNA ID s599 and s30515, respectively). Etoposide and MG132 were purchased from Sigma-Aldrich (Dorset, UK) as was PYR41 ( 4[4-(5-Nitro-furan-2-ylmethylene)-3,5-dioxo-pyrazolidin-1-yl]-benzoic acid ethyl ester).

#### TARDIS Assay.

**Fig. 1.**
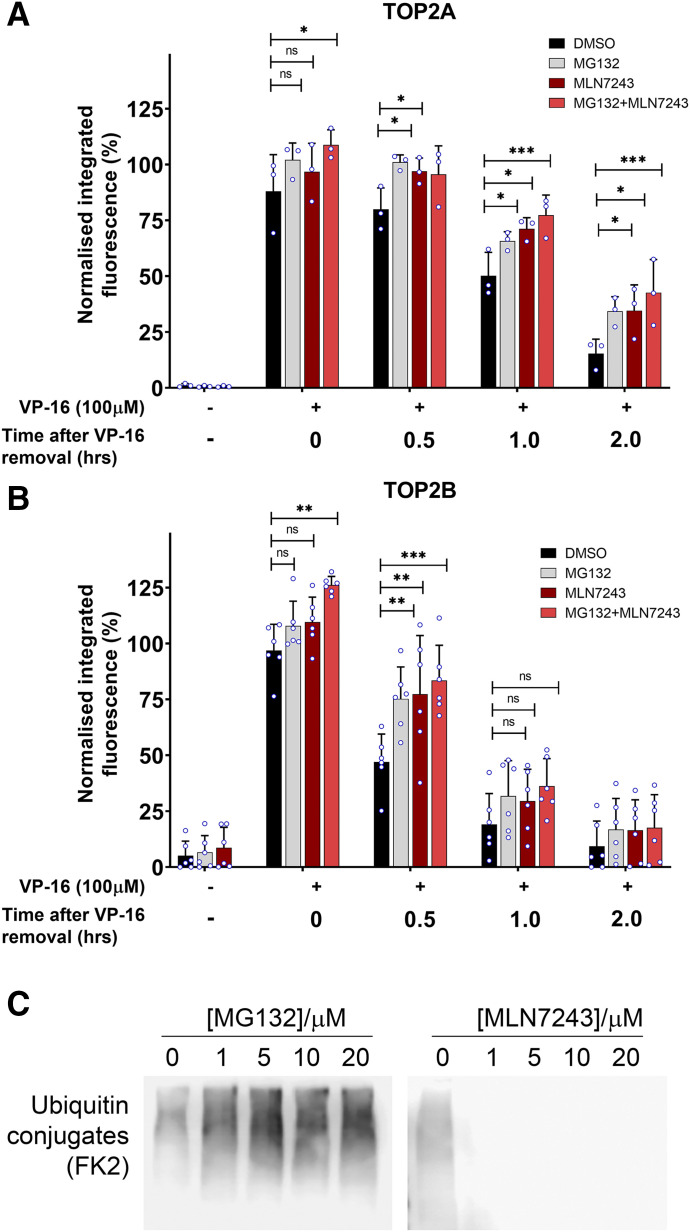
Effect of inhibiting E1 ubiquitin-activating enzyme on TOP2-DNA complex levels measured using the TARDIS assay. (A and B) K562 cells were treated for 2 hours with etoposide alone or in combination with 10 µM MG132, 10 µM MLN7243, or 10 µM MG132 and 10 µM MLN7243. Cells were incubated for up to a further 2 hours in etoposide-free medium but in the continued presence of DMSO, 10 µM MG132, or 10 µM MLN7243. Levels of TOP2A- and TOP2B-DNA complexes were measured 0, 0.5, 1, and 2 hours after etoposide removal using the TARDIS assay. Median normalized integrated fluorescence values of three independent experiments are shown (circle symbols) along with the mean ± S.D. of the medians. Statistical analyses were performed by two-way ANOVA with comparisons to etoposide-alone treatment using Dunett’s post hoc test. (C) K562 cells incubated with the indicated concentration of MG132 or MLN7243 for 2 hours. Western blots of whole-cell extracts were probed with monoclonal antibody FK2, which recognizes all conjugated ubiquitin (monoubiquinated and polyubiquitinated proteins). ns, not significant.

**Fig. 2.**
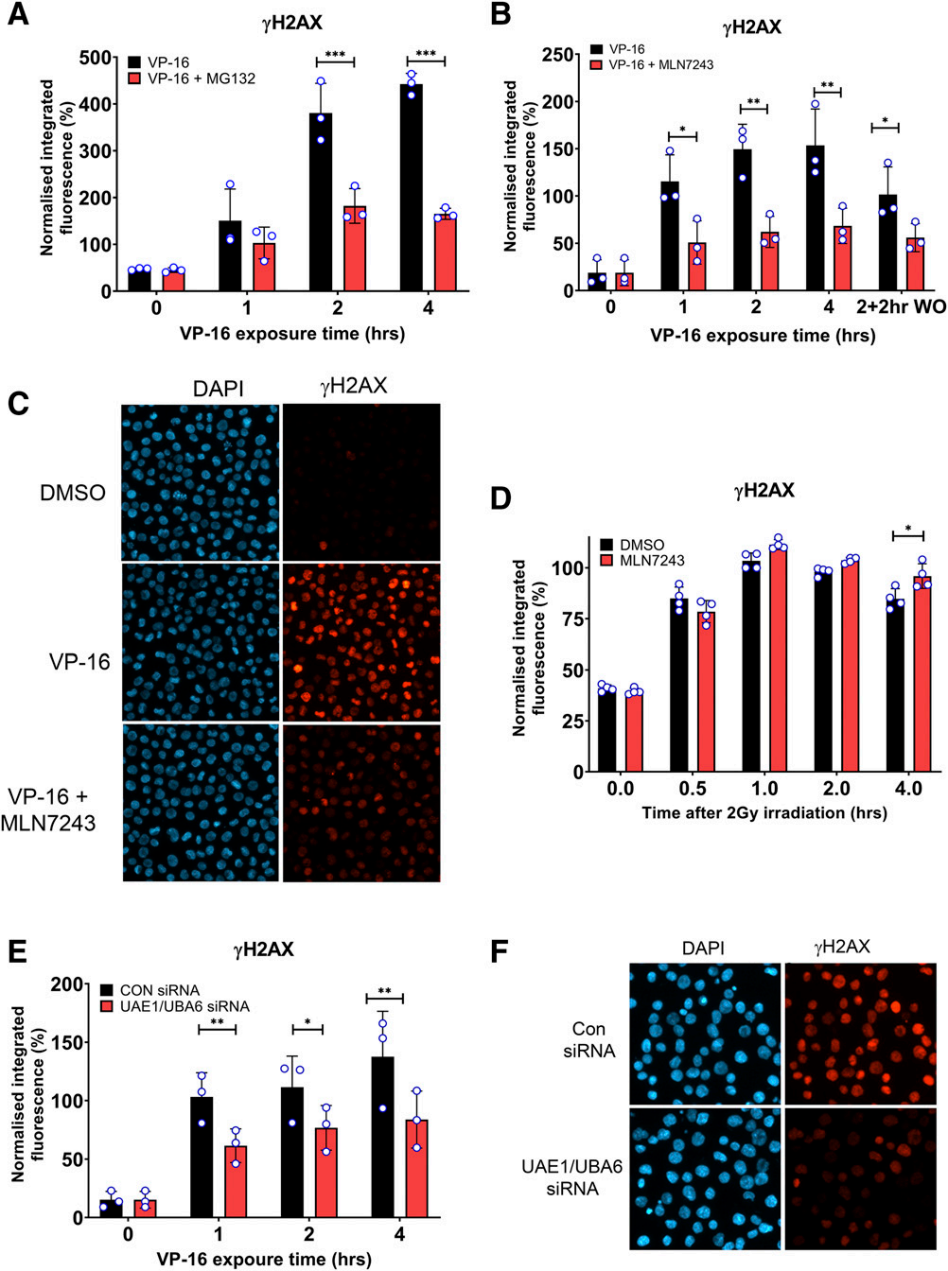
The appearance of etoposide-induced DSBs is reduced by chemical inhibition of E1 by inhibitor MLN7243 or by siRNA-mediated depeletion of E1 ubiquitin-activating enzymes. (A) K562 cells were treated with 10 µM etoposide (VP-16) alone or in combination with 10 µM MG132 for up to 4 hours, and protein-free DSBs were detected by γH2AX assay. Median-normalized integrated fluorescence for each replica experiment is indicated by small circle symbols, bars represent the means of the median values ±S.D. (B and C) The γH2AX assay was repeated in K562 cells treated with 10 µM VP-16 alone or in combination with 10 µM MLN7243 for up to 4 hours. Alternatively, cells were treated for 2 hours with etoposide followed by etoposide WO and 2 hours incubation in etoposide-free medium with or without MLN7243 (2 + 2 hours WO). (D) γH2AX levels were quantified after 2 Gy irradiation in the presence and absence of 10 µM MLN7243 and then normalized to a 1-hour postirradiation control. (E and F) γH2AX assay after siRNA silencing of UAE1 and UBA6. All values are normalized to a 1-hour 10 µM etoposide positive control. The medians from independent experiments are shown on each bar. Statistical significance was determined by two-way ANOVA (Bonferroni’s post hoc test). CON, control; DAPI, 4′,6-diamidino-2-phenylindole.

K562 cells were seeded at a density of 2 × 10^5^ cells/ml and incubated overnight before drug treatment. In experiments measuring the reduction of complexes after etoposide removal from the media, the signal at t_0_ needed to be high enough to generate an adequate signal-to-noise ratio. For this reason, cells were exposed to 100 μM etoposide for 2 hours. This is higher than the C_max_ in patient sera (Liston and Davis, 2017) but lower than the 250 μM etoposide used in Ban et al. (2013). TARDIS analyses were performed essentially as described previously (Willmore et al., 1998; Cowell and Austin, 2018; Cowell et al., 2019). TOP2 covalent DNA complexes were visualized by immunofluorescence using primary antibodies for TOP2 [4566-TOP2A and 4555-TOP2B, in-house antibodies raised to the C-terminal domain of human TOP2A and TOP2B, respectively (Atwal et al., 2019)] or ubiquitin (FK2, APU2, and APU3; Merck Millipore) and Alexa-488 or -594–coupled secondary antibodies (anti-rabbit A11008, or anti-mouse A11005; ThermoFisher Scientific, UK). Slides were counterstained with the DNA stain Hoechst 33258. Hoechst and AlexaFluor images were captured using an epifluorescence microscope (Olympus IX-81) fitted with an Orca-AG camera (Hamamatsu) and suitable narrow band filter sets employing a 10X objective. Slides were scored automatically as described previously (Atwal et al., 2019) using Volocity 6.3 software (PerkinElmer Inc., San Diego, CA). Data were subsequently represented, and statistical analysis was performed using GraphPad Prism 8.2 (Perkin Elmer). At least three replica experiments were performed for each TARDIS analysis. For each treatment, median integrated fluorescence values per nucleus were calculated, and these medians were then converted to a percentage of the median obtained for 100 μM etoposide. For Figs. 1, 2, and 5B, a separate 100-μM treatment was included with each replicate for normalization; for Fig. 5A, normalization was performed using the mean value of the medians obtained for 100-μM etoposide treatment alone. For bar charts, the mean ± S.D. of the mean of the median values was calculated.

#### *γ*H2AX Immunofluorescent Assays.

K562 cells were seeded at a density of 2 × 10^5^ cells/ml and incubated overnight. After drug treatment, cells were washed in PBS and allowed to adhere onto poly-L-lysine–coated microscope slides followed by fixation in 4% paraformaldehyde. Cells were permeabilized in KCM-T buffer (120 mM KCl, 20 mM NaCl, 10 mM Tris-HCl pH 8.0, 1 mM EDTA, 0.1% Triton X-100) and blocked overnight (KCM-T + 2% bovine serum albumin and 10% dried milk powder). Immunofluorescence staining was performed using anti–phospho-H2AX (Ser139) antibody (Merck Millipore) and Alexa 594–coupled secondary antibody [anti-mouse, A11005 prior to mounting with Vectashield with 4′,6-diamidino-2-phenylindole (Vector Laboratories, Burlingame, CA)]. Quantitative immunofluorescence and subsequent data analysis were performed as well as the TARDIS assay.

#### Western Blotting.

After drug treatment or siRNA knockdown, cells were washed in PBS and stored at −80°C until required. Whole cell extracts were prepared by SDS/DNase I extraction as previously described by Mirski et al. (1993), and Western blotting was performed by standard methods. Blots were probed with the following antibodies: anti-ubiquitin clone FK2 antibody (1:1000; Merck Millipore), APU2 anti-K48–linked ubiquitin (1:1000; Merck Millipore), anti-UAE1 (Abcam, Cambridge, UK), anti-UBA6 (ThermoFisher), or anti-actin (Abcam). Blots were developed on film or using the LI-COR C-DiGit Chemiluminescence Western Blot Scanner.

#### Growth Inhibition Assays.

Growth inhibition assays were performed in Nalm-6 cells because both a wild-type Nalm-6 cell line and a Nalm-6 cell line lacking TOP2B (Nalm-6^TOP2B−/−^) were available. It was suggested in a previous publication that PRT4165 increases the potency of teniposide specifically via TOP2A (Alchanati et al., 2009), and therefore, this allowed us to determine the effect of PRT4165 in cells expressing only TOP2A. Growth inhibition assays were carried out after 5 days continuous drug exposure, therefore low concentrations of drug needed to be used.

Cells were seeded in 96-well plates and incubated at 37°C, 5% CO_2_ for 24 hours prior to drug treatment (10,000 cells per well). Cells were then treated with varying concentrations of etoposide alone or in combination with a fixed concentration of the UAE inhibitor MLN7243 or BMI1 inhibitor PRT4165 and incubated for 120 hours. After 5 days of continuous drug exposure, 50 μl XTT reagent (50:1 XTT reagent to electron coupling reagent, XTT Cell Proliferation kit; Roche, UK) was added per well, and cells were incubated at 37°C for a further 4 hours. Absorbance values were obtained using the Bio-Rad 550 Microplate Reader (Bio-Rad) and analyzed using GraphPad Prism software (GraphPad Software), version 8. Growth inhibition values were determined by setting the values obtained with no drug as 100% for the etoposide-alone data and with MLN7243/PRT4165 alone as 100% for the etoposide plus MLN7243/PRT4165 data.

The IC_50_ values of etoposide alone versus IC_50_ of drug in combination with UAE or BMI1 inhibitor were used to calculate potentiation factor at 50% growth inhibition (Pf_50_). The inhibitory concentration of TOP2 poison in the presence of UAE or BMI1 inhibitor was divided by the concentration of TOP2 poison alone for each separate experiment.

#### Data Analysis.

Statistical analysis was performed using Graph Pad Prism 8. The details of tests performed are given in figure legends. Two-way ANOVA analysis was nonrepeated measures. For signifying *P* values, * refers to *P* < 0.05, ** refers to *P* < 0.01, *** refers to *P* < 0.001, and **** refers to *P* < 0.0001. Error bars in bar charts represent S.D. values. Sample sizes (numbers of replicate experiments) were specified in advance of data acquisition based on prior knowledge of the characteristics of the assays involved and anticipating occasional lost or failed samples. For Fig. 6, data are presented as growth curves with each point representing the mean ± S.E.M. from replica values.

## Results

### 

#### Effect of the UAE Inhibitor MLN7243 on Levels of TOP2-DNA Complexes.

To investigate the requirement for ubiquitination in the removal of etoposide-induced TOP2A- and TOP2B-DNA complexes, K562 cells were treated with the UAE inhibitor, MLN7243 (Misra et al., 2017; Hyer et al., 2018). The conjugation of the 76–amino acid protein ubiquitin to the target protein lysine occurs in a sequential manner involving an E1 UAE, an E2-conjugating enzyme, and an E3-ligating enzyme. The first step requires the activation of ubiquitin, which involves the formation of a high-energy thioester bond between ubiquitin and ubiquitin-activating enzyme (UAE1 or UBA6 in human cells) (Groettrup et al., 2008), and therefore, the E1 inhibitor MLN7243 inhibits all ubiquitination. The effect of UAE inhibition on the removal of etoposide-induced TOP2-DNA complexes was examined using the TARDIS assay.

Drug-stabilized TOP2A- and TOP2B-DNA complexes induced in cells can be visualized and quantified using the TARDIS assay, and the kinetics of removal of these complexes can be measured after drug washout. Once in drug-free media, some TOP2-DNA complexes are resealed by completion of the enzymes reaction cycle. Those complexes that are not spontaneously reversed require repair processes. Consistent with other studies (Mao et al., 2001; Zhang et al., 2006; Fan et al., 2008; Alchanati et al., 2009; Sunter et al., 2010), we previously demonstrated that efficient repair of TOP2 complexes on chromatin is partly dependent on proteasomal activity (Lee et al., 2016). This approach was used to address the role of ubiquitin in the processing of etoposide-induced TOP2-DNA complexes. In the TARDIS assay, drug-treated cells are mixed in agarose and spread onto microscope slides. The embedded cells are then lysed in buffer containing SDS and high salt, thus removing all noncovalently bound proteins and leaving behind only covalently bound TOP2 on genomic DNA in the presence of etoposide (Cowell et al., 2011). TOP2-DNA complexes are then visualized by immunofluorescence.

K562 cells were treated for 2 hours with 100 µM etoposide (VP-16) alone or in combination with 10 µM MLN7243 (a specific UAE inhibitor) (Misra et al., 2017; Hyer et al., 2018) or 10 µM of the proteasome inhibitor MG132. After 2 hours, the culture medium was removed and replaced with etoposide-free medium containing DMSO, MLN7243, or MG132 to maintain inhibition of ubiquitination or the proteasome, respectively. The TARDIS assay was used to measure levels of TOP2A- and TOP2B-DNA complexes after 2 hours continuous exposure to etoposide (0 hours after etoposide removal) and after 0.5, 1, and 2 hours incubation in etoposide-free medium.

As previously observed, TOP2A and TOP2B complex levels were both dramatically increased after 2 hours exposure to 100 µM etoposide compared with untreated cells. Neither MG132 nor MLN7243 resulted in TOP2-DNA complex formation on their own, nor did they significantly affect the accumulation of TOP2 complexes during the 2-hour continuous etoposide incubation (Fig. 1, A and B). Consistent with previous observations (Lee et al., 2016), etoposide-induced TOP2A- and TOP2B-DNA complex levels fell to less than 25% of the original levels 2 hours after etoposide removal. However, levels of remaining TOP2A-DNA complexes were higher in the presence of 10 µM MG132. This was statistically significant at 0.5, 1, and 2 hours after etoposide washout (*P* < 0.05). Remaining TOP2B-DNA complexes were significantly higher at 0.5 hours after etoposide removal in the presence of MG132 compared with cells treated with etoposide alone (*P* < 0.001). This is consistent with the shorter half-life of the TOP2B complexes after removal from drug-containing media (Willmore et al., 1998). Incubation of cells with 10 µM MLN7243 also slowed the removal of etoposide-induced TOP2A- and TOP2B-DNA complexes similarly to proteasome inhibition. Levels of remaining TOP2A-DNA complexes were significantly higher at 0.5, 1, and 2 hours after etoposide washout (*P* < 0.05, 0.001, and 0.001, respectively), and TOP2B-DNA complexes were significantly higher after 0.5 hours (*P* < 0.001). To determine whether the effects of MG132 and MLN7243 are epistatic, cells treated with etoposide were also cotreated with both MG132 and MLN7243. When both inhibitors were administered together, the levels of etoposide-induced TOP2A- and TOP2B-DNA complexes that accumulated during the 2-hour incubation were significantly increased (*P* < 0.01 and 0.001, respectively). However, no additive effect was observed on the rate of removal of TOP2A or TOP2B complexes when both inhibitors were administered together with etoposide compared with each inhibitor alone. This suggests that both inhibitors exert their effects via the same pathway and that the route to proteasomal degradation of etoposide-induced TOP2-DNA complexes is ubiquitin-dependent as it requires E1 ubiquitin-activating enzyme activity. Even in the presence of MG132 and/or MLN7243, the complex levels eventually return to background levels but more slowly. This suggests that the proteasomal repair pathway is probably not the only pathway for repair of the TOP2-DNA complexes. Attempts at estimating the proportion of TOP2-DNA complexes processed via a ubiquitin-dependent route are complicated by the fact that these complexes resolve after etoposide washout through the combined effect of one or more repair/processing pathways together with spontaneous reversal of the complexes by completion of the enzymes reaction cycle after removal of etoposide. From the data in Fig. 1, MLN7243 results in approximately 20% and 100% additional retention of TOP2A 1 and 2 hours after etoposide washout, respectively (in relation to the signal for each treatment at the time of drug washout). Thus, at later time points at least, a substantial proportion (up to 50%) of the removal of TOP2A complexes appears to be ubiquitin-dependent. The overall reversal rate is faster for TOP2B, and 30 minutes after etoposide washout, MLN7243 results in approximately 50% additional retention compared with the DMSO control. Thus, a substantial proportion (approximately one-third) of the disappearance of TOP2B complexes appears to be attributable to a ubiquitin-dependent mechanism.

Figure 1C shows Western blots probed with antibody FK2, which detects all ubiquitin conjugates. Inhibition of the proteasome by MG132 increased the amount of ubiquitin conjugates detected. In contrast, ubiquitinated conjugates were absent after inhibition with 10 µM MLN7243 (even in the presence of MG132, Fig. 4A), thus confirming potent inhibition of ubiquitination.

The specificity of MLN7243 for ubiquitin E1 enzymes was recently demonstrated by Misra et al. (2017), and thus the effect of MLN7243 on levels of TOP2-DNA complexes is unlikely to be due to off-target effects. Indeed, removal of TOP2A-DNA complexes was also decreased by another structurally distinct UAE inhibitor, PYR41 (Supplemental Fig. 1A). TARDIS experiments were performed after the cotreatment of cells with or without 50 µM PYR41. At this concentration, PYR41 reduced levels of ubiquitin conjugates even in the presence of MG132 (as measured by Western blotting, Supplemental Fig. 1B) and significantly reduced TOP2A complex resolution at 30 and 60 minutes after removal of etoposide (Supplemental Fig. 1A). For TOP2B, complex levels were significantly increased at t0 (2 hours treatment with etoposide) and remained significantly higher than the non-PYR41–treated cells 30 minutes after etoposide removal. To test the possibility that this effect was due to nonspecific inhibition of other ubiquitin-like E1 enzymes, the TARDIS assay was performed in the presence and absence of the highly specific Nedd8-activating enzyme inhibitor, MLN4924. Neddylation is required for the ubiquitination of proteins by a specific class of E3 ubiquitin ligases (the cullin-RING family). However, MLN4924 did not affect the levels of etoposide-induced TOP2-DNA complexes after removal of etoposide. The TARDIS data are shown as scatter plots in Supplemental Fig. 2 and suggest the effect of MLN7243 is not due to nonspecific inhibition of Nedd8-activating enzyme.

Furthermore, MLN7243 treatment did not affect levels of SUMOylated TOP2-DNA complexes as measured by TARDIS assay (Supplemental Fig. 3), thus indicating that MLN7243 does not inhibit SUMO-activating enzyme at the concentration used in this assay.

#### Ubiquitination Is Required for the Appearance of Etoposide-Induced DSBs.

The degradation of TOP2-DNA complexes by the proteasome leads to the appearance of protein-free DSBs, which are otherwise concealed by TOP2 protein and which result in S-139 phosphorylation of histone H2AX (Zhang et al., 2006; Zhao et al., 2006; Lyu et al., 2007; Fan et al., 2008). The specificity of H2AX phosphorylation for DSBs in this setting is supported by the previous observations that H2AX phosphorylation mirrors DSB break induction measured by other means (alkaline Comet or constant field gel electrophoresis) (Smart et al., 2008; Muslimović et al., 2009) and that the disappearance of etoposide-induced *γ*H2AX foci after treatment is delayed in cells treated with the DSB repair inhibitor NU7411 and in nonhomologous end joining–deficient DNA ligase 4 null cells (Riballo et al., 2004; Zhao et al., 2006). To determine whether the appearance of etoposide-induced DSBs is also dependent on E1 activity, the *γ*H2AX assay was used to measure levels of TOP2-free DSBs after etoposide treatment alone or in combination with the E1 inhibitor MLN7243 or the proteasome inhibitor MG132. As previously reported (Zhang et al., 2006; Fan et al., 2008; Tammaro et al., 2013), coincubation with MG132 reduced the appearance of the etoposide-induced *γ*H2AX signal (Fig. 2A), which was statistically significant after 2 and 4 hours of drug treatment (*P* < 0.001 and 0.001) where *γ*H2AX signal was reduced by approximately 50%. Notably, etoposide-induced *γ*H2AX levels remained significantly above background even in the presence of MG132, which was consistent with the presence of alternative proteasome-independent mechanisms of TOP2-DNA complex processing to DSBs.

*γ*H2AX levels were also reduced in the presence of the E1 inhibitor MLN7243 compared with etoposide alone (Fig. 2, B and C), and this was statistically significant at all time points tested (*P* < 0.05). In addition to continuous exposure to etoposide for 1, 2, or 4 hours, we also included a 2-hour etoposide treatment followed by etoposide removal and 2 hours incubation in etoposide-free medium or medium containing MLN7243 [2 + 2 hours washout (WO)]. This is equivalent to the 2-hour time point (t2) in the TARDIS assay in Fig. 1, and the 2-hour continuous exposure is equivalent to the t0 in the TARDIS assay in Fig. 1. To test whether the effect of UAE inhibition was specific to etoposide-induced DNA damage, the *γ*H2AX assay was performed after X-ray irradiation in the presence and absence of the E1 inhibitor MLN7243. Ionizing radiation-induced *γ*H2AX levels were not reduced in the presence of the E1 inhibitor MLN7243 (Fig. 2D). Conversely, *γ*H2AX levels were slightly increased by E1 inhibition 4 hours after irradiation (*P* < 0.01), which is consistent with inhibition of DNA repair (Moudry et al., 2012). This shows that ubiquitination is not required for the phosphorylation of histone H2AX but is involved specifically in the appearance of topoisomerase-mediated DSBs after etoposide exposure.

#### siRNA Knockdown of Ubiquitin-Activating Enzymes.

The role of ubiquitin in the removal of etoposide-induced TOP2-DNA complexes was also investigated after siRNA knockdown of UAE1 and UBA6. This did significantly reduce the appearance of etoposide-induced *γ*H2AX signal after continuous etoposide exposure, albeit to a lesser degree than was observed with MG132 or MLN7243 (Fig. 2, E and F). Phosphorylation of histone H2AX was still detectable after UAE1 and UBA6 knockdown, thereby reflecting other ubiquitin-independent mechanisms of TOP2-DNA complex removal or incomplete suppression of E1 activity by these siRNAs.

**Fig. 3.**
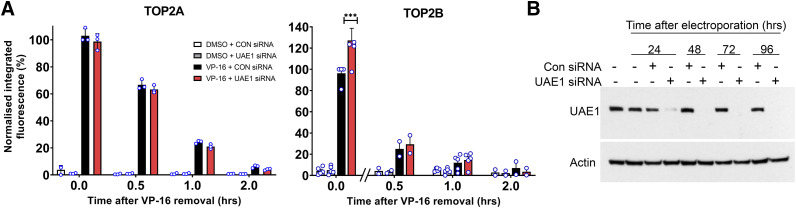
The effect of siRNA-mediated depletion of UAE1 on the processing of etoposide-induced TOP2-DNA complexes. (A) K562 cells were treated with UAE1 siRNA for 72 hours and then incubated for 2 hours with 100 µM VP-16 or 0.2% DMSO, which was followed by etoposide removal and a further 2 hours incubation in etoposide-free medium. Cells were collected at 0, 0.5, 1, and 2 hours after etoposide removal, and TOP2-DNA complex levels were quantified by TARDIS assay. Statistical comparisons were made by two-way ANOVA with Bonferroni’s post hoc test. (B) siRNA knockdown of UAE1 for 24, 48, 72, and 96 hours in K562 cells was tested by Western blot probing for UAE1.

The TARDIS assay was also performed on siRNA knockdown cells (UAE1 siRNA) to measure levels of etoposide-induced TOP2A- and TOP2B-DNA complexes compared with control cells transfected with nonsilencing siRNA (CON siRNA). Levels of TOP2B-DNA complexes were significantly increased in UAE1 siRNA knockdown cells after 2 hours of exposure to etoposide (*P* < 0.001, Fig. 3A). However, unlike E1 inhibition with MLN7243 (Fig. 1), levels of TOP2A-DNA complexes were not significantly affected by siRNA knockdown of the E1 ubiquitin-activating enzyme, UAE1 (Fig. 3A), despite efficient UAE1 silencing (Fig. 3B). In addition, TOP2A- and TOP2B-DNA complexes returned to background levels after the removal of etoposide, regardless of UAE1 knockdown. This could be due to residual UAE1 activity or the activity of the second and more recently discovered UAE, UBA6 (Groettrup et al., 2008). However, the removal of etoposide-induced TOP2-DNA complexes was also unaffected by UBA6 siRNA or double siRNA knockdown of UAE1 and UBA6 (Supplemental Fig. 4, A and B). The scatter plots shown in Supplemental Fig. 4 show the signal from individual cells, and the number of cells analyzed is shown above each column. The knockdown by UAE1 siRNA is shown in Fig. 3B, and the knockdown by UBA6 and the double knockdown are shown in Supplemental Fig. 4, C and D.

It is unclear why siRNA-mediated depletion of UAE significantly reduces levels of etoposide-induced H2AX phosphorylation but not the resolution of TOP2-DNA complexes observed using the TARDIS assay. However, several factors may contribute to this apparent discrepancy. Firstly, spontaneous reversal is a major contributor to complex resolution upon etoposide washout (see above). This could mask a small effect of UAE depletion on TOP2 complex processing as observed in the TARDIS assay. In contrast, H2AX phosphorylation occurs as a result of processing to protein-free breaks and so would be expected to be more sensitive to modest changes in processing efficiency. Secondly, the *γ*H2AX assay simultaneously measures the processing of both TOP2A and TOP2B complexes, whereas the TARDIS assay is isoform-specific. Thus, small differences with UAE knockdown may be more readily detectable in the *γ*H2AX assay because of the combined effects of both isoforms. Thirdly, although we measure loss of the original TOP2-DNA complex signal over a period of 2 hours using the TARDIS assay, the *γ*H2AX assay was used to quantify the accumulation of signal over time. So, at longer time points (1 or 2 hours) there was a reducing signal-to-noise ratio for the TARDIS assay but a robust and increasing signal for the H2AX assay. Notably, the 2 hours of etoposide incubation in the *γ*H2AX assays shown in Fig. 2E is equivalent to the 0-hour washout from the TARDIS experiments in Fig. 3A. At this time point, more TOP2B complexes were retained in the siRNA-depleted TARDIS sample, which is consistent with the reduction in H2AX signal observed in the siRNA-treated cells.

**Fig. 4.**
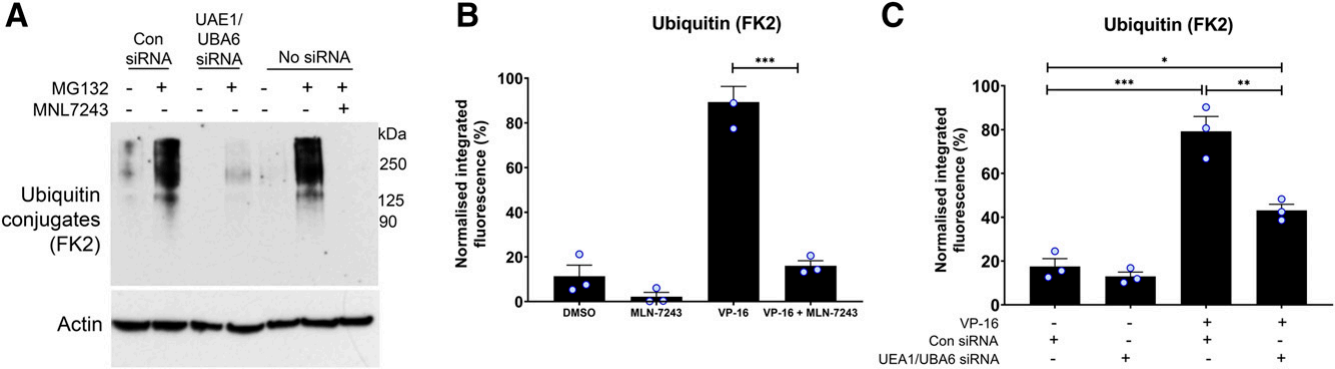
MLN7243 inhibits the ubiquitination of TOP2-DNA complexes. (A) K562 cells transfected with control siRNA, UAE1, and UBA6 siRNA or no siRNA were treated with 0.1% DMSO or 10 µM MG132 for 2 hours. Cells without siRNA were also treated with 10 µM MLN7243 to compare levels of ubiquitination activity with UAE1/UBA6 knockdown cells. Total ubiquitination levels were examined by Western blot and probing with clone FK2 antibody, which detects all conjugated ubiquitin. (B) K562 cells were treated with 100 µM etoposide alone or in combination with 10 µM of the UAE inhibitor MLN7243 for 2 hours, and levels of ubiquitinated TOP2-DNA complexes were measured using the TARDIS assay. (C) Control cells (CON siRNA) and double–UAE1/UBA6 siRNA knockdown cells were treated with 100 µM etoposide for 2 hours, and levels of ubiquitinated TOP2-DNA complexes were quantified using the TARDIS assay (*n* = 3); medians of independent experiments are shown as circles on the bar charts. Statistics was done using two-way ANOVA. Significance comparisons were made by by *t* test (B) or one-way ANOVA with Tukey’s post hoc test (C).

Differences between siRNA knockdown and small molecule inhibitor approaches may also be explained by incomplete knockdown of E1-activating activity with siRNA. To test the effectiveness of the siRNA knockdown on protein ubiquitination, a ubiquitination assay was performed to compare levels of remaining E1 activity in siRNA knockdown cells with MLN7243-treated cells. Despite efficient siRNA-mediated silencing of UAE1 and UBA6 E1 proteins determined by Western blotting (Fig. 3B; Supplemental Fig. 4, C and D), some ubiquitination activity remained detectable in UAE1/UBA6 siRNA-treated cells, as evidenced by the presence of ubiquitinated proteins in whole cell extracts by Western blotting with antibody FK2 (Fig. 4A). Upon treatment with a proteasome inhibitor, there was an accumulation of ubiquitinated proteins that would otherwise be degraded by the proteasome. This was evident in control cells treated with MG132 for 2 hours compared with the DMSO control (Fig. 4A, compare lane 1 with lane 2 and lane 5 with lane 6). Although the MG132-induced accumulation of ubiquitinated proteins was reduced in UAE1/UBA6 knockdown cells (Lane 4), it was not eliminated. In contrast, there was no detectable accumulation of ubiquitinated proteins in MLN7243-treated cells (lane 7), which was consistent with complete E1 inhibition. Thus, under the conditions employed, ubiquitination activity was more robustly inhibited by chemical inhibition of UAE with MLN7243 than by UAE1/UBA6 siRNA knockdown.

#### Investigating TOP2 Ubiquitination Using the TARDIS Assay.

The role of ubiquitin in the removal of TOP2-DNA complexes could involve the direct ubiquitination of TOP2 or the modification of other proteins involved in processing. To investigate this further, ubiquitination of the TOP2 trapped in DNA complexes was examined by TARDIS assay.

The TARDIS assay was adapted to study the post-translational modification of TOP2-DNA complexes by probing with anti-ubiquitin antibodies. During TARDIS slide processing, cells were lysed in buffer containing 1% SDS and 1 M NaCl, which removes all noncovalently bound proteins from DNA. This includes histones and other proteins tightly associated with chromatin, such as RNA polymerase II and Ku80, which is confirmed by probing TARDIS slides for histones, RNA polymerase II, and KU antigen (Supplemental Fig. 5). Ubiquitin TARDIS detected ubiquitin on the TOP2, which is covalently bound to DNA in TOP2-DNA complexes. Levels of conjugated ubiquitin on the TOP2-DNA covalent complexes were measured using the FK2 antibody in TARDIS, and the signal was detectable after 2 hours etoposide exposure (Fig. 4, B and C; Supplemental Fig. 6). This demonstrated that TOP2-DNA complexes are ubiquitinated. Levels of ubiquitin conjugates were reduced to background levels when cells were coincubated with E1 inhibitor MLN7243 (Fig. 4B). Levels of ubiquitinated TOP2-DNA complexes were also reduced in UAE1/UBA6 siRNA knockdown cells but remained significantly above background levels (Fig. 4C), which was consistent with the siRNA knockdown of E1 activity being incomplete as shown in Fig. 4A. Notably, these differences were not due to reduced levels of TOP2A- or TOP2B-DNA complexes, as neither MLN7243 treatment nor UAE1/UBA6 siRNA knockdown reduced levels of TOP2-DNA complexes after 2 hours continuous etoposide exposure when measured by TARDIS assay (see Figs. 1 and 3; Supplemental Fig. 4, respectively).

TARDIS slides were also probed with linkage-specific ubiquitin antibodies that detect K48- and K63-linked polyubiquitin chains. Low levels of both K48- and K63-linked ubiquitin were detected in etoposide-treated cells (Supplemental Fig. 6), which are typically associated with proteasomal degradation and signaling pathways, respectively.

#### Use of the TARDIS Assay to Study the Role of BMI1/RING1A E3 Ubiquitin Ligase in the Processing of Etoposide-Induced TOP2-DNA Complexes.

**Fig. 5.**
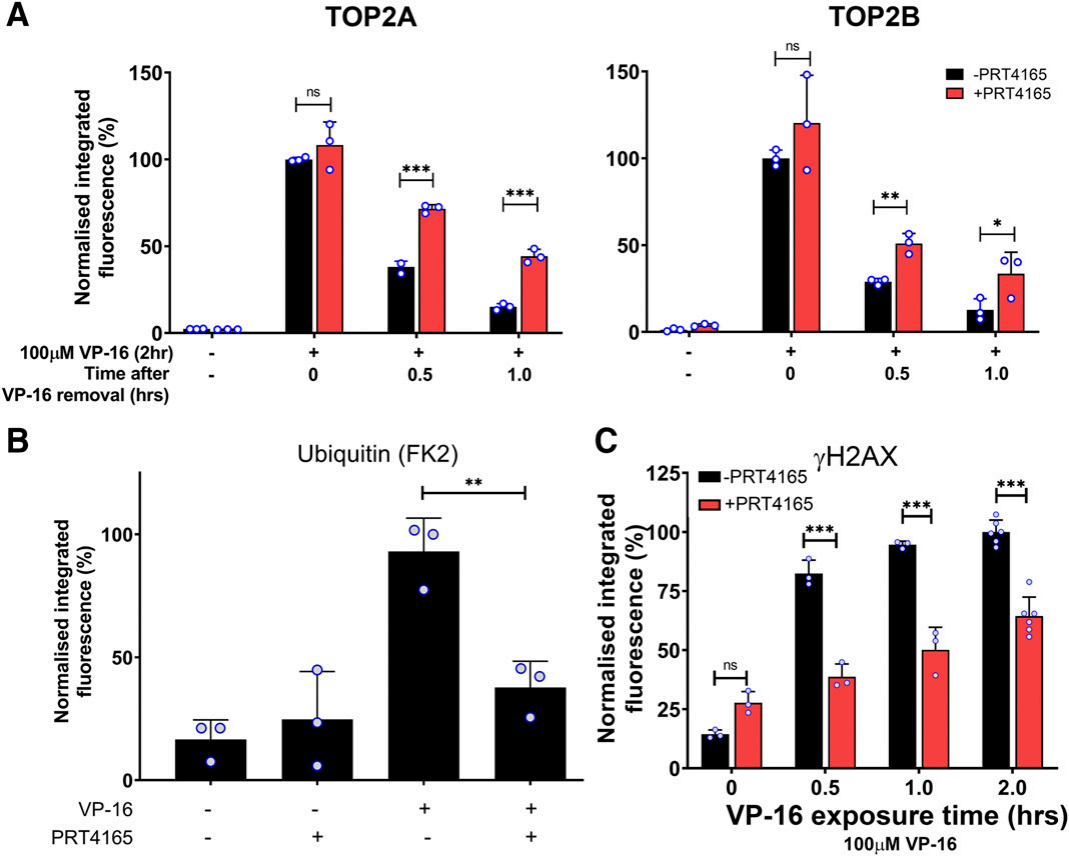
BMI1/RING1A is involved in the processing of TOP2-DNA complexes to protein-free DSBs. (A) K562 cells were treated with 100 µM etoposide (VP-16) alone or in combination with 90 µM PRT4165 for 2 hours. Cells were incubated in etoposide-free medium containing DMSO or 90 µM PRT4165 to maintain inhibition of BMI1/RING1A for 0, 0.5, 1, or 2 hours, and levels of TOP2A- and TOP2B-DNA complexes were measured using the TARDIS assay. Averages were normalized to the 2-hour continuous 100 µM etoposide control (0 hours after VP-16 removal), and statistical comparisons were made by two-way ANOVA with Bonferroni’s post hoc test (B) K562 cells were treated with 100 µM etoposide alone or in combination with 90 µM PRT4165 for 2 hours. Cells were collected and processed as per the TARDIS assay, and slides were probed with anti-ubiquitin antibody clone FK2. Averages were normalized to a 2-hour 100 µM etoposide control, and statistical comparisons were made by one-way ANOVA. (C) Cells were treated continuously with 100 µM etoposide alone or in combination with 90 µM PRT4165 for up to 4 hours, and levels of protein-free DSBs were measured using the ɣH2AX assay. Average integrated fluorescence values were normalized to the mean 1-hour 100 µM etoposide treatment value, and statistical comparisons were made by two-way ANOVA with Bonferroni’s post hoc test. Medians of independent experiments are shown as circles on the bar charts. ns, not significant.

BMI1/RING1A is an E3 ubiquitin ligase previously implicated in the teniposide-induced proteasomal degradation of TOP2A-DNA complexes (Alchanati et al., 2009). In the current study, the role of BMI1/RING1A in the processing of etoposide-induced TOP2-DNA complexes was investigated using the TARDIS assay and the BMI1/RING1A inhibitor, PRT4165 (Alchanati et al., 2009; Ismail et al., 2013). K562 cells were treated with 100 µM etoposide alone or in combination with 90 µM PRT4165 for 2 hours, and this was followed by incubation in etoposide-free medium containing DMSO or PRT4165 to maintain inhibition of BMI1/RING1A. Cells were collected 0, 0.5, 1, and 2 hours after etoposide removal, and levels of TOP2A- and TOP2B-DNA complexes were measured using the TARDIS assay. Levels of TOP2A- and TOP2B-DNA complexes were not significantly affected after 2 hours continuous exposure to etoposide (Fig. 5A). However, levels of remaining TOP2A-DNA complexes were significantly higher in the presence of PRT4165 after 30 or 60 minutes incubation in etoposide-free medium (*P* < 0.001), which was consistent with a role for BMI1/RING1A in the processing of TOP2A-DNA complexes. Levels of remaining TOP2B-DNA complexes were also significantly higher in PRT4165-treated cells 30 and 60 minutes after etoposide removal (*P* < 0.05). This suggests that BMI1/RING1A is involved in the removal of both TOP2A- and TOP2B- complexes from DNA.

To test the effect of PRT4165 on the ubiquitination of TOP2-DNA complexes, the ubiquitin TARDIS assay was performed in K562 cells treated with 100 µM etoposide alone or in combination with 90 µM PRT4165. As shown in Fig. 5B, levels of etoposide-induced ubiquitinated TOP2-DNA complexes were significantly reduced in the presence of PRT4165, suggesting BMI1/RING1A is involved in the ubiquitination of TOP2.

The effect of PRT4165 on the processing of etoposide-induced TOP2-DNA complexes was also investigated using the *γ*H2AX assay. K562 cells were treated with 100 µM etoposide alone or in combination with 90 µM PRT4165 for up to 4 hours, and DSB levels were measured after 0, 1, 2, and 4 hours continuous drug exposure. The appearance of etoposide-induced DSBs was significantly reduced in the presence of PRT4165 at all time points tested (*P* < 0.001, Fig. 5C), suggesting the ubiquitin-dependent processing of etoposide-induced TOP2-DNA complexes to DSBs is largely BMI1/RING1A-dependent.

#### Effect of UAE Inhibition on the Growth-Inhibitory Effects of Etoposide.

To investigate the effect of UAE inhibition on the growth-inhibitory effects of the TOP2 poison etoposide, growth inhibition assays (XTT) were first used to determine the concentration of inhibitor giving 20% growth inhibition (IC_20_) of MLN7243. For Nalm-6 cells, the IC_20_ of MLN7243 was 400 nM.

To examine the effect of MLN7243 on the growth-inhibitory effects of etoposide, Nalm-6 wild-type cells were treated with increasing concentrations of etoposide alone or in combination with 400 nM MLN7243. The effect of MLN7243 on the growth-inhibitory effects of each TOP2 poison was quantified by a potentiation factor (Pf_50_), which was calculated as a ratio of the IC_50_ of TOP2 poison alone and the IC_50_ of TOP2 poison in combination with MLN7243. Potentiation was deemed statistically significant if there was a significant difference between the IC_50_ of TOP2 poison alone versus IC_50_ of TOP2 poison in combination with MLN7243, as determined by an unpaired *t* test. Strikingly, coincubation of Nalm-6 cells with MLN7243 significantly reduced the IC_50_ of etoposide (*P* < 0.001), resulting in a Pf_50_ of 3.1.

To investigate the role of TOP2B in the potentiation of etoposide with MLN7243, growth inhibition assays were repeated in Nalm-6^*TOP2B−/−*^ cells. Nalm-6^*TOP2B−/−*^ cells were treated with increasing concentrations of etoposide for 120 hours, alone or in combination with 400 nM MLN7243. MLN7243 significantly reduced the IC_50_ of etoposide from 169 to 104.33 nM in the presence of MLN7243, giving a Pf_50_ of 1.63 (*P* = 0.0001). The Pf_50_ value for etoposide was significantly smaller in Nalm-6^*TOP2B−/−*^ cells compared with the Pf_50_ value of 3.1 in Nalm-6 wild-type cells. This suggests that the potentiation of MLN7243 is mediated by both TOP2A and TOP2B.

#### Effect of BMI1/RING1A Inhibition on the Growth-Inhibitory Effects of TOP2 Poison Etoposide.

**Fig. 6.**
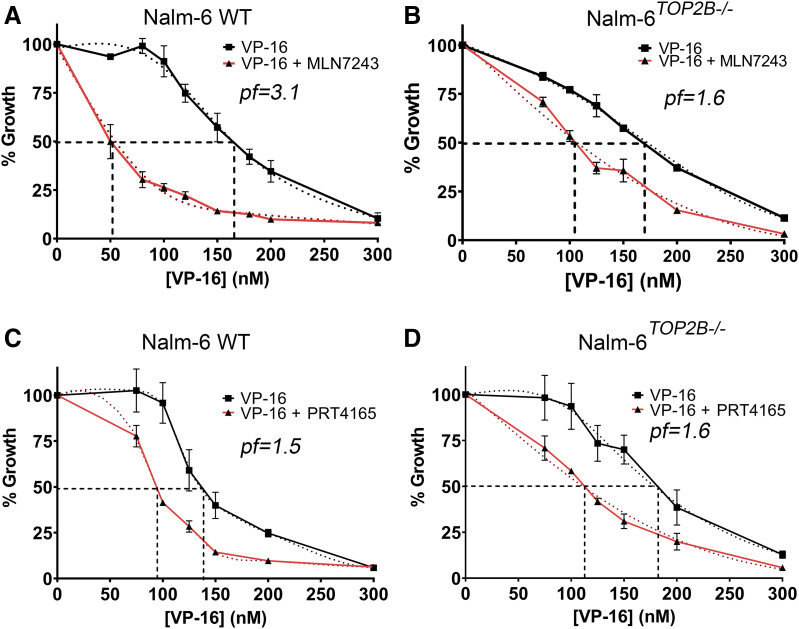
Inhibition of ubiquitin-activating enzyme or BMI1/RING1A sensitizes cells to etoposide. Nalm-6 (A) or Nalm-6^*TOP2B−/−*^ (B) cells were incubated with increasing concentrations of etoposide (VP-16) alone or in combination with 400 nM MLN7243 for 120 hours. Growth inhibition was determined by XTT assay. IC_50_ values were determined by plotting dose-response curves; resulting *Pf*_*50*_ values (the fold difference in IC_50_ values) are shown on the plots. Data plotted are the mean values from at least three separate experiments ±S.E.M. Nalm-6 (C) or Nalm-6^*TOP2B−/−*^ (D) cells were incubated with increasing concentrations of etoposide (VP-16) alone or in combination with 35 µM PRT4165 for 120 hours. Growth inhibition, IC_50_, and *Pf*_*50*_ values were determined (A and B). WT, wild type.

PRT4165 is a small molecule inhibitor of the E3 ubiquitin ligase BMI1/RING1A, which was shown to inhibit the teniposide-induced degradation of TOP2A-DNA complexes and the autoubiquitination of BMI1/RING1A (Alchanati et al., 2009) and which, as we have demonstrated, also inhibits the processing of both TOP2A and TOP2B DNA complexes (Fig. 5, A and C). The IC_20_ of PRT4165 was determined to be 35 μM for Nalm-6 cells. Incubation of cells with PRT4165 at this concentration significantly reduced the IC_50_ of etoposide (*P* < 0.05) with a Pf_50_ value of 1.5. This shows that the growth-inhibitory effect of etoposide can be increased by inhibition of the BMI1/RING1A ubiquitin ligase. To examine the role of each TOP2 isoform in the potentiation of TOP2 poisons with PRT4165, growth inhibition assays were also performed in Nalm-6^*TOP2B−/−*^ cells. PRT4165 potentiation of etoposide remained significant (*P* < 0.05) with a Pf_50_ of 1.6 in Nalm-6^*TOP2B−/−*^ cells. The very similar Pf_50_ values observed in wild-type and TOP2B null Nalm-6 cells suggest that potentiation of etoposide by PRT4165 operates largely via TOP2A. This is consistent with the finding of Alchanati et al. (2009), whereby BMI1/RING1A silencing reduced drug-induced TOP2A degradation but was not predicted from the finding that PRT4165 inhibits the processing of both TOP2A and TOP2B complexes (Fig. 5). This apparent inconsistency could be explained by the different conditions under which the assays were performed. For growth inhibition assays, cells were exposed continuously for 5 days to etoposide up to 300 nM (i.e., several times the IC_50_ value), whereas for TOP2 complex reversal assays (TARDIS) cells were exposed over a shorter course to a much higher etoposide concentration (100 μM, necessary to induce a robust signal for quantification of complex reversal). Thus, a caveat of this study is that the conditions for measuring complex reversal and cell sensitization were not equivalent. We hypothesize that under the conditions used for growth inhibition assays, PRT4165 sensitized cells to etoposide largely via inhibition of TOP2A-DNA complex processing, which is consistent with the TOP2A-specific effect reported by Alchanati et al. (2009), whereas in TARDIS assays we were able to detect an effect on both TOP2 isoforms, perhaps because of the much larger accumulation of TOP2-DNA complexes. However, it should be noted that in the Alchanati et al. (2009) study, the cells were incubated in drug-free media for 30 minutes prior to the detection of complexes, and the TOP2B complex half-life is shorter than that of TOP2A. Notably, the degree of potentiation is similar for PRT4165 and MLN7243 in TOP2B null cells (Fig. 6, B and D). This suggests that under growth inhibition conditions, the effect of MLN7243, but not PRT4165, is significantly dependent on TOP2B, which is consistent with a general effect on proteasomal processing of TOP2 complexes resulting from E1 inhibition but a more specific effect on TOP2A with BMI1/RING1A inhibition.

## Discussion

The repair of drug-stabilized TOP2-DNA complexes is particularly challenging because of the covalent attachment of TOP2 to DNA. Among other mechanisms, TOP2 can be removed from the TOP2-DNA complex by proteasomal degradation (Mao et al., 2001; Zhang et al., 2006; Fan et al., 2008; Lee et al., 2016), and the remaining 5′-phosphotyrosine adduct may then be hydrolyzed by the 5′-phosphodiesterase, TDP2 (Cortes Ledesma et al., 2009; Zeng et al., 2011; Schellenberg et al., 2012; Gao et al., 2014). This culminates in the liberation of protein-free DNA ends that result in a DNA damage response, including phosphorylation of histone H2AX (Gittens et al., 2019; Gothe et al., 2019), and are a substrate for nonhomologous end joining repair (Mårtensson et al., 2003; Malik et al., 2006; Maede et al., 2014). Ubiquitination has been reported to play a role in the removal of stalled TOP2 complexes on DNA.

Ubiquitin itself contains seven lysine residues that can be ubiquitinated, forming polyubiquitin chains (Komander, 2009). The conjugation of ubiquitin to target proteins requires multiple enzymatic steps, which firstly involve an E1 ubiquitin-activating enzyme (UAE1 or UBA6), then an E2-conjugating enzyme, and finally an E3-ligating enzyme. The key first step is the activation of ubiquitin, which involves the formation of a high-energy thioester bond between ubiquitin and ubiquitin-activating enzyme (UAE1 or UBA6 in human cells) (Groettrup et al., 2008). Thus, the UAE small-molecular inhibitor MLN7243 inhibits all ubiquitination. Previous studies on the role of E1 in the processing of TOP2-DNA complexes have used a murine cell line with a temperature-sensitive E1; however, two studies using this cell line reported different results regarding its role in the processing of epipodophyllotoxin-induced TOP2-DNA complexes. Both previous studies used Western blotting to determine the levels of TOP2 under various conditions, which allows only the measurement of pooled protein populations, including both unbound TOP2 and TOP2 in TOP2-DNA complexes in a manner that is difficult to quantify. While data in the first study suggested that the proteasomal degradation of TOP2B was ubiquitin-dependent, the second study proposed a ubiquitin-independent mechanism of TOP2B proteasomal degradation after etoposide treatment, involving the collision of drug-stabilized TOP2B-DNA complexes with elongating RNA polymerase II. In this model, the proteasome was suggested to be recruited to the trapped TOP2B-DNA complex by RNA polymerase II–associated 19S AAA ATPases (Ban et al., 2013). Thus the requirement for ubiquitin in the degradation of TOP2B-DNA complexes has remained unclear (Mao et al., 2001; Ban et al., 2013).

A major aim of the current study was therefore to clarify inconsistences in the literature regarding the role of the E1 ubiquitin-activating enzyme in the removal of TOP2 complexes after drug exposure. To do this, a combination of small molecule inhibitor and siRNA knockdown approaches were used. Supraclinical concentrations of etoposide (100 µM) were used to ensure our data were comparable to previous studies in which concentrations of up to 250 µM etoposide were used. High concentrations of etoposide were also required to generate a robust signal in the TARDIS assay, which would otherwise become undetectable and unquantifiable with time after etoposide removal from the cell culture media. Although the use of high etoposide concentrations was necessary to address the aims of this study, it is important to note that circulating concentrations of etoposide in clinical use are much lower than 100 µM. A C_max_ of 33 µM is cited in a review by Liston and Davis (2017) and, depending upon the regimen, much lower concentrations of etoposide may be present in patient sera. Lower etoposide concentrations (up to 0.3 µM) were used in the 5-day growth inhibition assays shown in Fig. 6.

The effect of UAE inhibition by MLN7243 on the removal of etoposide-induced TOP2-DNA complexes was examined using the TARDIS assay, thereby allowing quantitative measurement of TOP2-DNA complex removal on a cell-by-cell basis (Fig. 1; Supplemental Fig. 1). Inhibition of UAE reduced the removal of etoposide-stabilized TOP2-DNA covalent complexes to a similar degree as proteasomal inhibition with MG132, which is consistent with a role for ubiquitination in the removal of TOP2 complexes. Indeed, combination experiments with MLN7243 and MG132 suggest that the ubiquitin-dependent pathway is epistatic with the proteasomal processing pathway. In addition, processing of the TOP2-DNA complexes to frank DSBs (detectable by *γ*H2AX assay) was also inhibited by MLN7243 and comparable to the effect of inhibition of the proteasome by MG132, further suggesting that they are part of the same pathway (Fig. 2, A and B). Processing of TOP2-DNA complexes to DSBs was also reduced by siRNA knockdown of E1 enzymes (Fig. 2, E and F). As alluded to above, there have been conflicting reports regarding the requirement for protein ubiquitination in the resolution of TOP2 poison–induced TOP2-DNA complexes (Mao et al., 2001; Ban et al., 2013). However, the experiments reported here employing pharmacological inhibition and siRNA knockdown of UAE activity strongly support a ubiquitin-dependent component to the processing of TOP2-DNA covalent complexes to DSBs that evoke a DNA damage response.

We have shown that ubiquitin is required for efficient processing of TOP2-DNA complexes to protein-free DSBs, and protein ubiquitination is therefore an important layer of regulation in the repair of TOP2 poison–induced DNA damage. We also show that at least some etoposide-induced TOP2-DNA complexes are conjugated with ubiquitin. However, an important limitation of the ubiquitin TARDIS assay is that it does not reveal the ubiquitination status of TOP2 protein that is not covalently bound to DNA (before the addition of etoposide). Therefore, we were not able to determine whether the observed TOP2 ubiquitination occurs as a consequence of TOP2 poisoning or represents a constitutive level of TOP2 ubiquitination. Similarly, although the simplest explanation for the ubiquitin dependence of TOP2-DNA complex processing is a requirement for conjugation of ubiquitin to TOP2, we cannot exclude alternative explanations, including the ubiquitination of another protein that is required for efficient TOP2-DNA complex processing.

It is also important to note that, like UAE inhibition, proteasomal inhibition is reported to deplete levels of nuclear ubiquitin (Xu et al., 2004; Dantuma et al., 2006; Heidelberger et al., 2018). Therefore, we also cannot fully exclude the possibility that the observed proteasome-dependent processing of TOP2-DNA complexes is due to inhibition of another ubiquitin-dependent (but proteasome-independent) pathway.

BMI1/RING1A is an E3 ubiquitin ligase previously implicated in the proteasomal degradation of teniposide-induced TOP2A-DNA complexes (Alchanati et al., 2009). We show here that, like UAE inhibition, inhibition of BMI1/RING1A also reduces the processing of etoposide-induced TOP2A-DNA complexes. We also show that BMI1/RING1A is required for the efficient processing of TOP2B-DNA complexes, indicating a ubiquitin-dependent processing pathway that is common to both TOP2 isoforms. However, as described above, the ubiquitin- and BMI1/RING1A-dependent processing of TOP2-DNA complexes may involve the ubiquitination of TOP2, or alternatively, the modification of another protein involved in TOP2-DNA complex repair. Although PRT4165 inhibited processing of both TOP2A- and TOP2B-DNA complexes, growth inhibition experiments in wild-type and TOP2B null Nalm-6 cells indicated that etoposide potentiation by PRT4165 was largely independent of TOP2B. However, it is important to note that for practical reasons the TARDIS and growth inhibition assays used very different etoposide concentrations (100 µM vs. ≤300 nM, respectively). These data also allow the possibility of at least one other E3 ubiquitin ligase that targets TOP2B, whose contribution may be more significant at lower etoposide concentrations.

Given the clinical interest in the ubiquitin-proteasome system and the ongoing development of specific inhibitors, these results suggest that the therapeutic cytotoxicity of TOP2 poisons could be enhanced through combination therapy with UAE inhibitors or by specific inhibition of the BMI1/RING1A ubiquitin ligase, which would lead to increased cellular accumulation or persistence of TOP2-DNA complexes. In support of this idea, we have shown previously that proteasomal inhibition potentiates the cytotoxicity of TOP2 poisons in a cell culture system (Lee et al., 2016). In the present study we show that coincubation of etoposide with MLN7243 or PRT4165 potentiates the cytotoxicity of etoposide (Fig. 6). In addition, we hypothesize that reduced conversion of TOP2-DNA complexes to protein-free DNA DSBs may also diminish the occurrence of genotoxic side effects of TOP2 poisons, including the formation of leukemia-inducing chromosome translocations.

## Abbreviations

DSB: double-strand break
H2AX: histone H2A family member X
*γ*H2AX: S-139 phospho-histone H2AX
IC_20_: concentration at 20% growth inhibition
Pf_50_: potentiation factor at 50% growth inhibition
siRNA: small interfering RNA
SUMO: small ubiquitin-like modifier
TARDIS: Trapped in Agarose DNA Immunostaining
TOP2: DNA topoisomerase II
TOP2A: DNA topoisomerase II*α*
TOP2B: DNA topoisomerase II*β*
UAE: ubiquitin-activating enzyme
WO: washout
